# Editorial: Community series in towards precision medicine for immune-mediated disorders: advances in using big data and artificial intelligence to understand heterogeneity in inflammatory responses, volume III

**DOI:** 10.3389/fimmu.2026.1878718

**Published:** 2026-06-02

**Authors:** Xu-jie Zhou, Le-ting Zhou, Celine C. Berthier, Swapan K. Nath

**Affiliations:** 1 Renal Division, Peking University First Hospital, Beijing, China; 2 Department of Nephrology, The Affiliated Wuxi People’s Hospital of Nanjing Medical University, Wuxi Medical Center, Nanjing Medical University, Wuxi People’s Hospital, Wuxi, China; 3 Division of Nephrology and Hypertension, Department of Medicine, Mayo Clinic, Rochester, MN, United States; 4 Department of Internal Medicine, Division of Nephrology, University of Michigan, Ann Arbor, MI, United States; 5 Arthritis and Clinical Immunology Research Program, Oklahoma Medical Research Foundation, Oklahoma City, OK, United States

**Keywords:** artificial intelligence, big data, heterogeneity, immune-mediated disorders, precision medicine & genomics

The “Towards Precision Medicine for Immune-Mediated Disorders” Community Series was conceived to address a central challenge in immunology: why patients who share broad clinical labels such as lupus, rheumatoid arthritis, psoriasis or inflammatory bowel disease can display strikingly different inflammatory profiles, trajectories and treatment responses. Earlier volumes highlighted how large-scale cohorts, multi-dimensional omics data and modern computational frameworks could be combined to reveal disease-associated pathways and to begin mapping this heterogeneity across populations.

This third volume moves the field further along that trajectory. Across eight articles, the Topic brings together work spanning cardiovascular immunology, inflammatory skin disease, inflammatory bowel disease, systemic autoimmunity and neuro-immune disorders. Collectively, these contributions illustrate how genetic and molecular profiling, quantitative bibliometrics, high-dimensional biomarker discovery and data-driven modelling can be used not only to characterize individual diseases in depth, but also to place them on shared axes of immune dysregulation that cut across traditional specialty boundaries.

## Mapping immune-mediated disorders along shared genetic axes

A major goal of this Series has been to move beyond single-disease silos and to define the structure of the immune-mediated disease spectrum from a genetic perspective. In this volume, Fominykh et al. analyze genome-wide association study summary statistics for fifteen polygenic immune-mediated disorders using genomic structural equation modelling, local genetic correlation analyses and causal mixture modelling. Their work supports a four-factor continuum extending from predominantly autoimmune diseases to predominantly autoinflammatory conditions, with mixed entities occupying intermediate positions.

Within this framework, systemic lupus erythematosus, primary Sjögren’s syndrome and systemic sclerosis cluster at an “autoimmune” pole characterized by strong adaptive immune components. At the other extreme, Crohn’s disease, ulcerative colitis and primary sclerosing cholangitis group together as an “inflammatory gut” factor with a prominent mucosal and barrier component. Rheumatoid arthritis, myasthenia gravis, autoimmune thyroid disease and psoriasis bridge these poles, illustrating how overlapping genetic architectures can underlie distinct clinical syndromes. By integrating global and locus-specific genetic correlations, and by linking these to tissue and pathway enrichment, this work offers a data-driven scaffold for rethinking how immune-mediated disorders are classified and compared.

Complementing this broad view, Wu et al. provide a comprehensive review of inflammatory bowel disease that reconstructs the immune-cellular regulatory network from susceptibility genes to cellular effectors and clinical phenotypes. They synthesize findings from genome-wide association studies, fine-mapping and functional genomics with single-cell and spatial transcriptomics to show how IL-23–Th17 signaling, autophagy pathways (including *NOD2* and *ATG16L1*), epithelial barrier regulators (such as *HNF4A* and *SLC* family transporters) and neuro-immune–metabolic axes collectively shape disease heterogeneity in Crohn’s disease and ulcerative colitis. This framework supports a shift from purely anatomic or phenotype-based classification to mechanistic endo-typing, in which patients are stratified by underlying immunogenetic circuits that may predict prognosis and therapeutic response.

## Multi-omics and biomarker discovery for autoimmune diseases

Another recurrent theme of the Series has been the development of robust biomarkers that can capture complex pathophysiology more faithfully than traditional single laboratory parameters. Zhang et al. present an extensive review of multi-omics-driven biomarker discovery across a broad spectrum of autoimmune diseases, including systemic lupus erythematosus, rheumatoid arthritis, type 1 diabetes, multiple sclerosis, vasculitis and others. They systematically describe how genomics, epigenomics, transcriptomics, proteomics, metabolomics and microbiomics have each contributed novel candidates and, more importantly, how integrated panels derived from these layers can overcome the modest sensitivity, specificity and dynamic range of conventional biomarkers such as anti-dsDNA, ANCA, rheumatoid factor or complement levels. The review emphasizes that multi-omics-based signatures are particularly valuable for three use cases. First, they can aid earlier and more accurate diagnosis, for example by using blood-based transcriptional or methylation markers in childhood-onset lupus, or urine proteomics in lupus nephritis. Second, they can support dynamic monitoring of disease activity and organ involvement, including prediction of flares before clinical deterioration. Third, they can inform treatment decisions by identifying molecular predictors of response or toxicity to targeted therapies, thereby supporting more individualized therapeutic strategies. Importantly, the authors also discuss how machine-assisted feature selection and network analysis are becoming integral to distilling high-dimensional omics data into actionable biomarker panels, while highlighting the need for rigorous validation in prospective, multi-center cohorts.

Jiang et al. approach biomarker development and mechanistic insight from a different angle, using bibliometric methods to analyze 1,469 publications on macrophage-associated immune regulation in atherosclerosis. Their analysis identifies leading authors, institutions and journals, but, crucially, it also reveals evolving research hotspots: autoantibody production and loss of tolerance; macrophage activation and polarization; immunometabolism, including itaconate and sirtuin pathways; and nuclear receptor signaling. By quantifying how the field has moved from traditional risk factors toward macrophage-centered immune regulation, this work underscores that the immune pathobiology of atherosclerosis now offers fertile ground for multi-omics and data-enabled precision approaches similar to those being developed in classic autoimmune diseases.

## Data-driven modelling of clinical and inflammatory profiles

Several articles in this volume illustrate how contemporary statistical and computational techniques can extract clinically meaningful patterns from population-level and clinical datasets. Yang et al. make use of more than 22,000 adults from the National Health and Nutrition Examination Survey to benchmark multiple classification models for identifying psoriasis based on simple blood-derived inflammatory indices, including neutrophil-to-lymphocyte ratio, systemic immune-inflammation index, monocyte-to-lymphocyte ratio and related composite markers. After careful handling of class imbalance and complex survey design, they show that gradient boosting models achieve the most favorable discrimination, and that monocyte-to-lymphocyte ratio and closely related indices consistently emerge as the most informative predictors. The absolute predictive performance of single indices remains modest, as one would expect a complex disease partly driven by genetic and environmental triggers. Nevertheless, this work provides an instructive example of how existing national survey data, when combined with flexible modelling and robust resampling strategies, can be repurposed to evaluate inexpensive, readily measurable markers for potential use in large-scale screening or risk stratification. It also highlights that even simple hematological parameters, when analyzed together rather than in isolation, can recapitulate elements of systemic inflammatory burden relevant to immune-mediated skin disease.

More broadly, Zhang et al. argue that such data-driven models will have their greatest impact when they are embedded within multi-omics pipelines and explicitly linked to longitudinal patient trajectories. They discuss how network-based and multivariate approaches can connect genetic variants, epigenetic marks, transcripts, proteins, metabolites, microbiome features and immune-cell phenotypes, and how causal inference and carefully designed validation studies are needed to distinguish mere correlates from candidate drivers and therapeutic targets. This perspective echoes the initial vision of the Community Series: to use large-scale, heterogeneous data to move from association to mechanism-informed prediction and, ultimately, to clinically relevant decision support.

## From disease-specific insight to shared priorities

**Figure 1:**
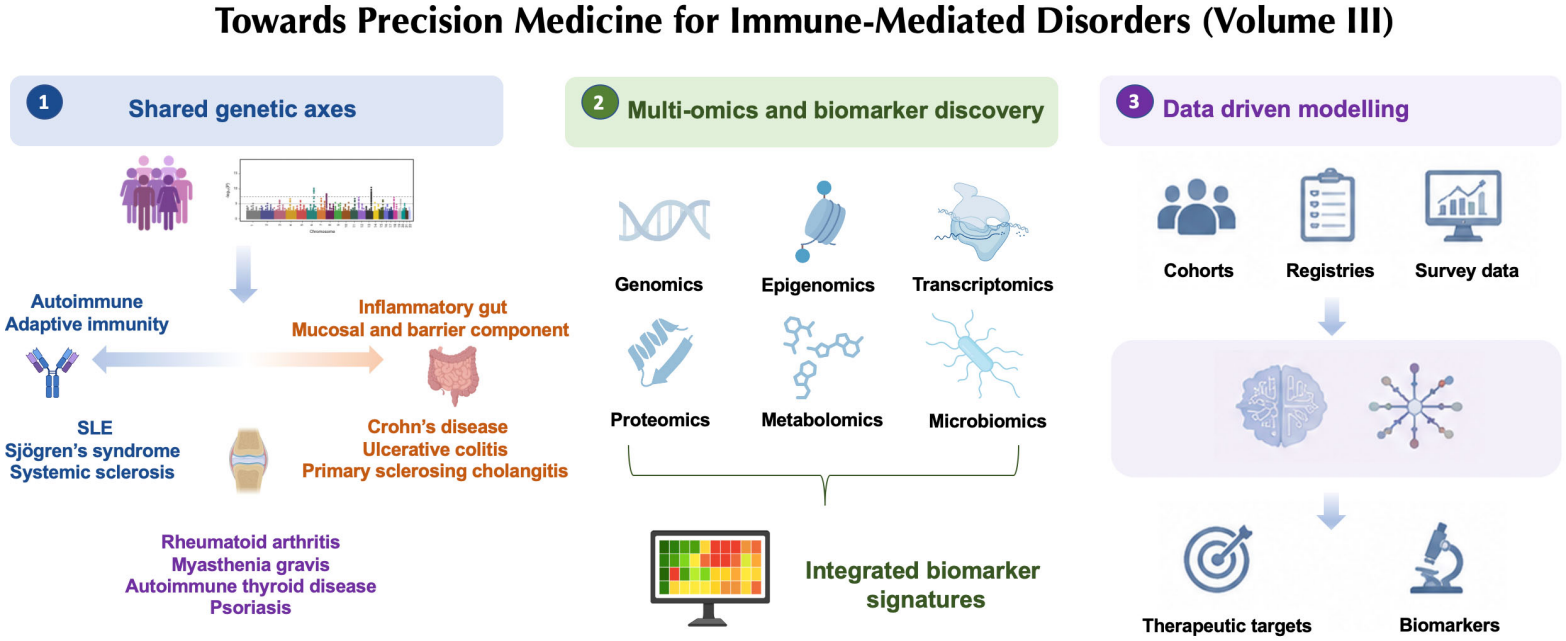
Precision medicine framework for immune-mediated disorders. This schematic illustrates three key advances discussed in this editorial: shared genetic and mechanistic axes for reclassifying immune-mediated diseases, multi-omics-based biomarker discovery, and data-driven modelling of clinical and inflammatory profiles. Together, these approaches support a shift from disease-specific silos toward integrated, mechanism-informed and data-enabled precision medicine.

Taken together, the eight articles in this volume advance the agenda of the Community Series in three important ways. First, they demonstrate that immune-mediated disorders can be meaningfully organized along shared genetic and mechanistic axes, rather than being treated as entirely distinct entities defined solely by organ involvement or traditional clinical labels. Second, they show that multi-omics-driven biomarker discovery is maturing from isolated candidate markers toward integrated signatures that can be tailored to specific clinical questions, from early diagnosis to monitoring and treatment selection. Third, they illustrate how contemporary analytic techniques applied to existing cohorts, registries and survey data can reveal patterns not obvious to conventional analyses and can help prioritize which markers and pathways warrant deeper experimental and clinical investigation (**Figure 1**).

Despite these advances, several methodological limitations warrant honest acknowledgment. Many contributions in this volume, consistent with the broader field, rely on cross-sectional designs, homogeneous study populations, and retrospective cohorts, limiting causal inference and generalizability. Omics-derived biomarker signatures, however statistically compelling, have a well-documented tendency to underperform in independent replication cohorts. Findings here should be interpreted with that caveat in mind. Machine learning models, including the boosting approaches highlighted by Yang et al., require prospective validation in clinically representative populations. Methodological choices, such as class imbalance correction, need systematic evaluation across diverse datasets to ensure that reported performance metrics are robust and generalizable. Acknowledging these constraints is not a counsel of pessimism but a necessary step toward designing the next generation of studies with greater rigor and translational intent.

Looking ahead, several priorities emerge. Methodologically, there is a need for scalable, interpretable analytical frameworks that can integrate diverse data types—genomes, transcriptomes, proteomes, metabolomes, microbiomes, imaging and longitudinal health records—while explicitly accounting for ancestry, sex and environmental context. Biologically, dissecting the temporal dynamics of immune networks, particularly within tissue-resident and stromal compartments, will be essential to understand why similar genetic risk profiles can lead to divergent disease trajectories. Clinically, prospective, multi-center studies are required to test whether omics- and data-guided stratification truly improves outcomes, reduces toxicity and enables more equitable access to precision care across global populations.

By assembling these contributions, Volume III consolidates and extends the trajectory set by the first two volumes (1, 2)—from conceptual framing and early technical demonstrations to integrated, cross-disease analyses and translationally oriented biomarker and modelling work. We anticipate that the frameworks, datasets and hypotheses presented here will catalyze further collaboration between immunologists, computational scientists and clinicians, and will help to bring precision medicine for immune-mediated disorders closer to everyday clinical practice (3–5).

## References

[1] Zhou XJ Macleod AS Tsoi LC . Editorial: advances in using big data and artificial intelligence to understand heterogeneity in inflammatory responses. Front Immunol. (2022) 13:948885. doi: 10.3389/fimmu.2022.948885. PMID: 35812380 PMC9258302

[2] Zhou X Laouar Y Tsoi LC . Editorial: community series in towards precision medicine for immune-mediated disorders: advances in using big data and artificial intelligence to understand heterogeneity in inflammatory responses, volume II. Front Immunol. (2025) 16:1553004. doi: 10.3389/fimmu.2025.1553004. PMID: 39877132 PMC11769832

[3] Zhou XJ Zhong XH Duan LX . Integration of artificial intelligence and multi-omics in kidney diseases. Fundam Res. (2023) 3:126–48. doi: 10.1016/j.fmre.2022.01.037. PMID: 38933564 PMC11197676

[4] Ong JCL Ning Y Collins GS Bitterman DS Beecy AN Chang RT . International partnership for governing generative artificial intelligence models in medicine. Nat Med. (2025) 31:2836–9. doi: 10.1038/s41591-025-03787-4. PMID: 40588674

[5] Altucci L Badimon L Balligand J Baumbach J Catapano AL Cheng F . Artificial intelligence and network medicine: path to precision medicine. NEJM AI. (2025) 2:10.1056/aira2401229. doi: 10.1056/aira2401229. PMID: 40918693 PMC12410635

